# Supplementary material for: Rational Design of Ni-Doped V2O5@3D Ni Core/Shell Composites for High-Voltage and High-Rate Aqueous Zinc-Ion Batteries
Source: Materials (Basel). 2023 Dec 30;17(1):215. doi: 10.3390/ma17010215 (PMC10779517; doi:10.3390/ma17010215)
Supplement: Supplementary file 1 [file materials-17-00215-s001.zip › materials-2768624-supplementary.pdf]

Rational Design of Ni-Doped V_2O_5@3D Ni Core/Shell Composites for High-Voltage and High-Rate Aqueous Zinc-Ion Batteries

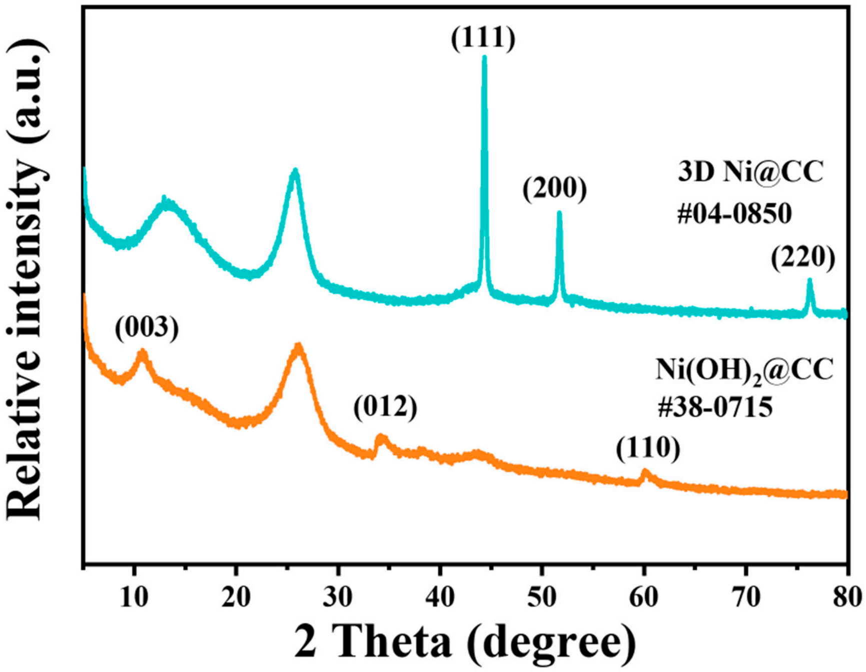

Figure S1. XRD patterns of Ni(OH)$_2$@CC and 3D Ni@CC.

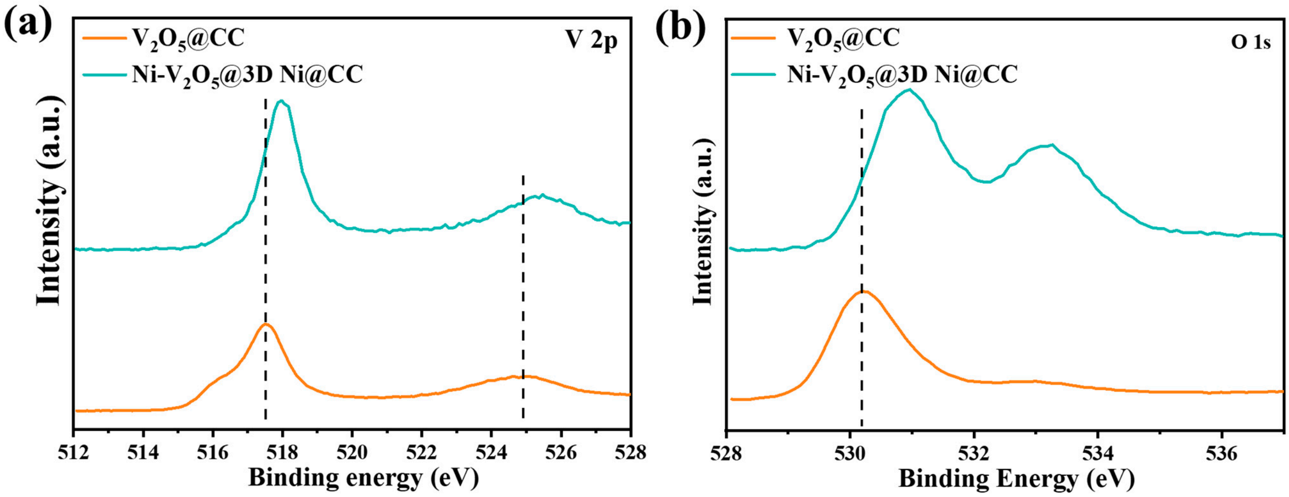

Figure S2. XPS spectra for 3D Ni@CC and Ni-V_2O_5@3D Ni@CC samples of (a) V and (b) O characteristic peaks.

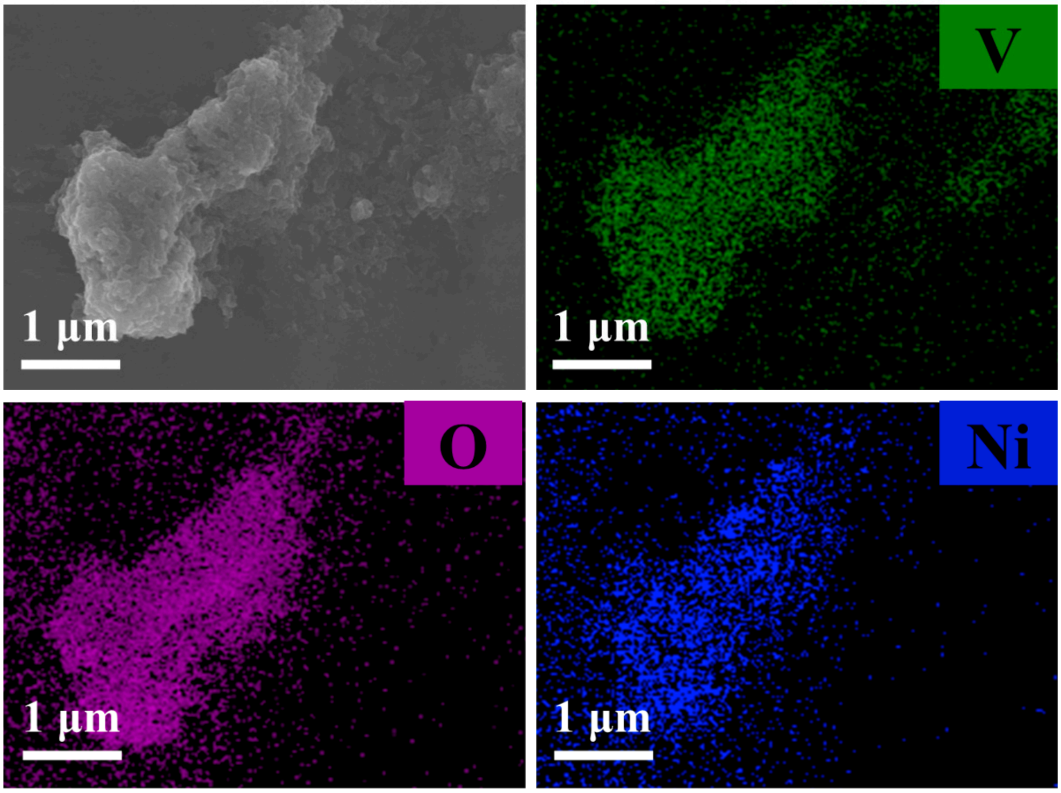

Figure S3. SEM image and corresponding elemental distribution of Ni-V_2O_5@3D Ni@CC.

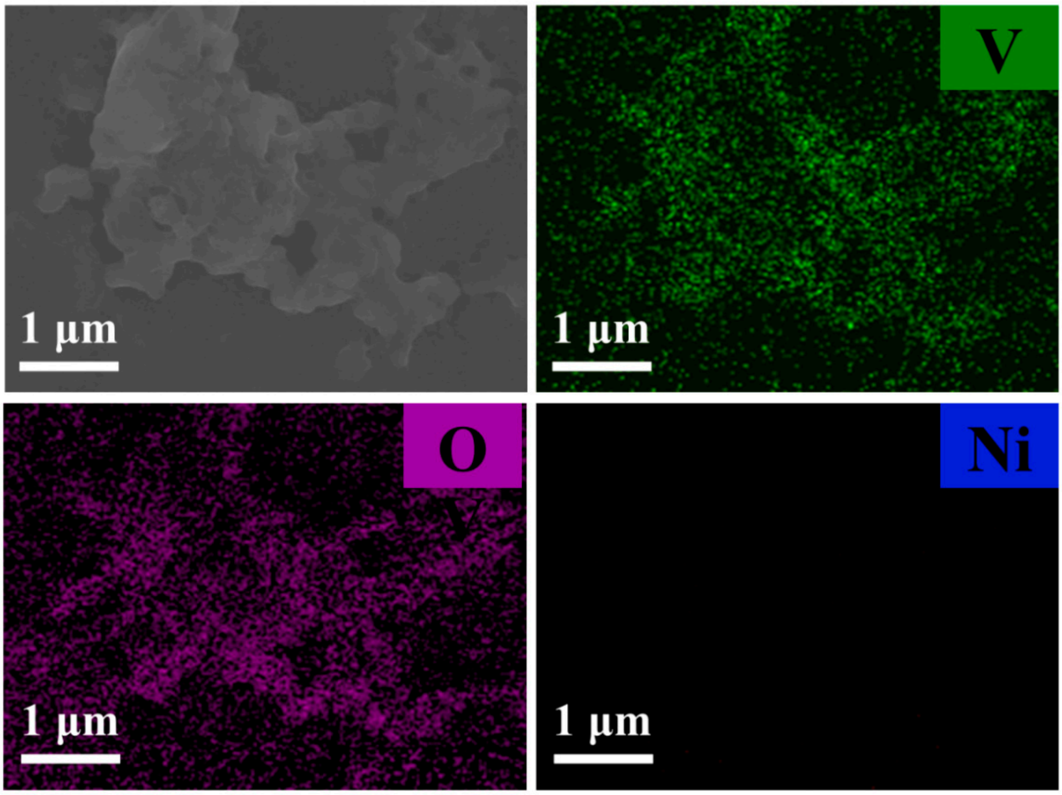

Figure S4. SEM image and corresponding elemental distribution of V_2O_5@CC.

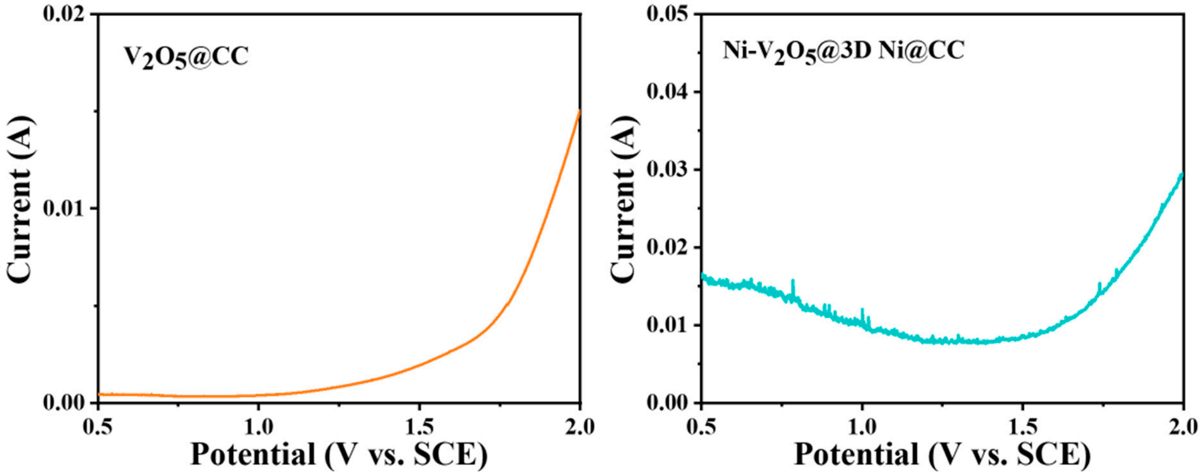

Figure S5. OER polarization curves of V_2O_5@CC and Ni-V_2O_5@3D Ni@CC.

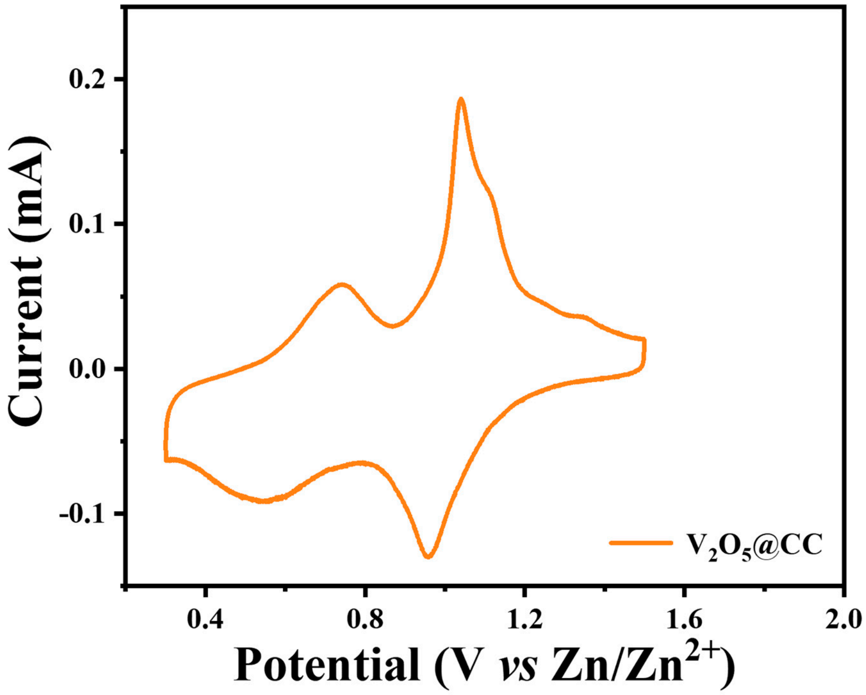

Figure S6. CV curves of V_2O_5@CC//Zn cell.

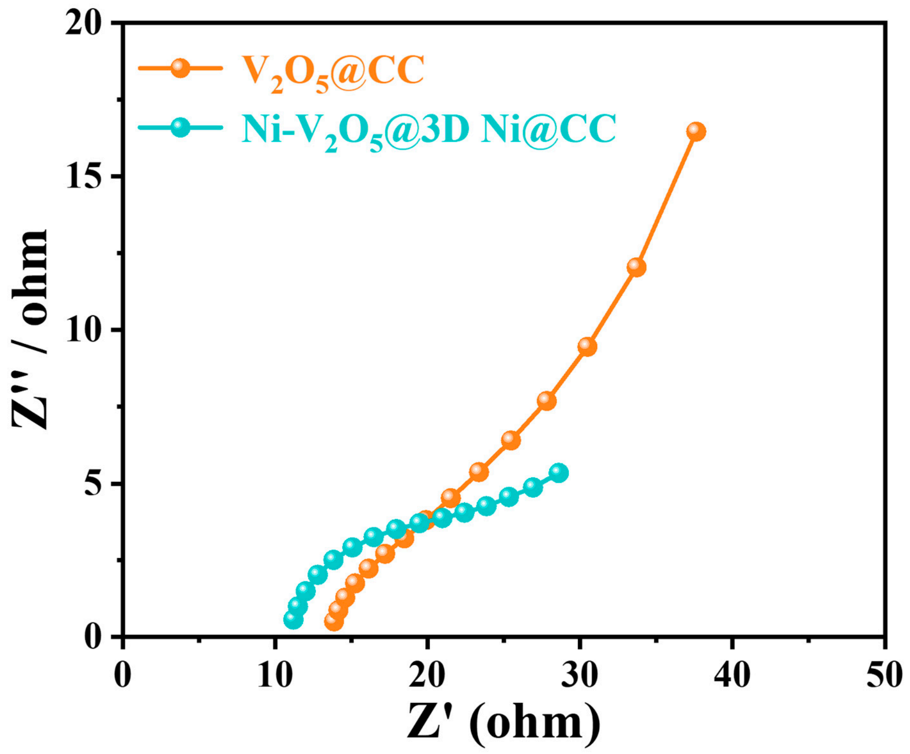

Figure S7. EIS Nyquist plots for V_2O_5@CC//Zn and Ni-V_2O_5@3D Ni@CC//Zn cells.
